# SBXception: A Shallower and Broader Xception Architecture for Efficient Classification of Skin Lesions

**DOI:** 10.3390/cancers15143604

**Published:** 2023-07-13

**Authors:** Abid Mehmood, Yonis Gulzar, Qazi Mudassar Ilyas, Abdoh Jabbari, Muneer Ahmad, Sajid Iqbal

**Affiliations:** 1 Department of Management Information Systems, College of Business Administration, King Faisal University, Al Ahsa 31982, Saudi Arabia; aafzal@kfu.edu.sa; 2 Department of Information Systems, College of Computer Sciences and Information Technology, King Faisal University, Al Ahsa 31982, Saudi Arabia; 3 College of Computer Science and Information Technology, Jazan University, Jazan 45142, Saudi Arabia; 4 Department of Human and Digital Interface, Woosong University, Daejeon 34606, Republic of Korea

**Keywords:** skin lesions, cancers, skin cancer, deep learning, machine learning

## Abstract

**Simple Summary:**

Skin cancer is a major concern worldwide, and accurately identifying it is crucial for effective treatment. we propose a modified deep learning model called SBXception, based on the Xception network, to improve skin cancer classification. Using the HAM10000 dataset, consisting of 10,015 skin lesion images, the model achieved an impressive accuracy on a test set. SBXception also showed significant improvements, requiring fewer parameters and reducing training time compared to the original model. This study highlights the potential of modified deep learning models in enhancing skin cancer diagnosis, benefiting society by improving treatment outcomes.

**Abstract:**

Skin cancer is a major public health concern around the world. Skin cancer identification is critical for effective treatment and improved results. Deep learning models have shown considerable promise in assisting dermatologists in skin cancer diagnosis. This study proposes SBXception: a shallower and broader variant of the Xception network. It uses Xception as the base model for skin cancer classification and increases its performance by reducing the depth and expanding the breadth of the architecture. We used the HAM10000 dataset, which contains 10,015 dermatoscopic images of skin lesions classified into seven categories, for training and testing the proposed model. Using the HAM10000 dataset, we fine-tuned the new model and reached an accuracy of 96.97% on a holdout test set. SBXception also achieved significant performance enhancement with 54.27% fewer training parameters and reduced training time compared to the base model. Our findings show that reducing and expanding the Xception model architecture can greatly improve its performance in skin cancer categorization.

## 1. Introduction

Among other cancers, skin cancer is considered one of the deadliest diseases. Around 1.2 million people died in 2020 due to skin cancer only [1]. According to the WHO [1], skin cancer was one of the most common cancers in terms of new cases in 2020, and the number of new cases is increasing dramatically [2,3]. One of the common causes of skin cancer is exposure of the skin to UV (ultraviolet) rays directly coming from the sun [4]. It is said that such rays affect fair-skinned people and those with sensitive skin more than dark-skinned ones [5].

Most deaths are caused by invasive melanoma, which constitutes only 1% of total skin cancer cases. From historical data, it is found that melanoma skin cancer cases are rising rapidly. According to the most recent report from the American Cancer Society, which provides data up until 2022 [6], it was estimated that approximately 99,780 cases of melanoma cancer would have been diagnosed by the end of the year, with 57,180 cases among men and 42,600 cases among women. The report also indicated that around 7650 deaths were expected due to melanoma cancer, with approximately 5080 deaths among men and 2570 deaths among women.

To cure any cancer, it is best to detect it at an early stage, and skin cancer is no different. Any unusual growth or new/changing skin spots must be evaluated. If there are any new lesions or any change in a lesion’s appearance, whether in size, color, or shape, it should be shown to a doctor and evaluated accordingly. To detect skin cancer, doctors use multiple techniques, and one of the ways is visual detection [7]. A manual has been developed by the American Center for the Study of Dermatology, and is used by doctors for initial screening. This manual is called asymmetry, border, color, and diameter (ABCD). At the initial stage, the doctor suspects a skin lesion on the patient’s body and recommends going for a biopsy [8]. The reports are examined, and a thorough check is performed to detect whether it is benign or malignant and the type of cancer [9]. Another technique, called dermoscopy, can be used to diagnose skin cancers [10]. In this technique, bright images of the skin lesion are captured, highlighting dark spots [11]. Nevertheless, these methods are inefficient because they cannot help diagnose the nature of the lesion. This is due to many reasons such as the presence of blood vessels or hair around the lesion, the intensity of the light, failure to correctly capture the shape of the lesion, and not identifying cancerous and non-cancerous lesions correctly [12,13,14].

The average accuracy of diagnosing skin cancers by manually examining dermoscopic images is 60% to 80%. The accuracy varies from one dermatologist to another based on their years of experience. It has been claimed that a dermatologist with three to five years of experience can have an accuracy of around 60%, whereas there is an improvement in accuracy by 20% if the dermatologist has 10+ years of experience [15]. Therefore, it can be claimed that dermoscopy requires extensive training to yield better diagnosis. Different types of skin cancers can be identified with the help of dermoscopic images. However, there are two main types of skin cancers: melanocytic and nonmelanocytic. Melanotic skin cancers consist only of melanoma and melanocytic nevi. However, nonmelanocytic skin cancers have many types, such as dermatofibroma (DF), vascular (VASC), benign keratosis lesions (BKL), basal cell carcinoma (BCC), and squamous cell carcinoma (SCC) [16].

Melanoma is a type of skin cancer arising from abnormal melanin production in melanocyte cells. It is the most prevalent and lethal form of skin cancer and is categorized into benign and malignant types [17]. While benign melanoma lesions contain melanin in the epidermal layer, malignant melanomas display excessive melanin production. The United States reports over five million new cases of skin cancer each year, with melanoma accounting for three-quarters of all skin cancer fatalities, resulting in 10,000 deaths annually [18]. In 2021, the US registered 106,110 cases of melanoma, leading to 7180 fatalities, with projections indicating a 6.5% increase in melanoma-caused deaths in 2022. In 2022, it is expected that 197,700 new cases of melanoma will be diagnosed in the US alone [19]. Every year, around 100,000 new cases of melanoma are discovered throughout Europe [20]. Melanoma is detected in 15,229 people in Australia each year [18,21]. Skin cancer incidence rates have climbed in the last decade, with melanoma rates increasing by 255% in the United States and 120% in the United Kingdom since the 1990s [22,23]. Melanoma, however, is considered a highly curable cancer if detected early. In the early stages, survival rates exceed 96%. In the advanced stage, by contrast, survival rates drop to 5%. When melanoma has spread throughout the body, treatment becomes more difficult [16].

The adoption of artificial intelligence (AI) and deep learning [24,25] has resulted in significant advancements in the accuracy and efficiency of skin cancer classification, assisting in the disease’s early diagnosis and treatment [26]. When trained on massive datasets of skin scans, AI systems may learn to recognize the characteristics of malignant cells and distinguish them from benign cells with high accuracy. Several studies have explored using AI and deep learning for skin cancer classification [27,28]. Khan et al. [29] adopted the DarkeNet19 model and trained it on multiple datasets such as HAM10000, ISBI2018, and ISBI2019. They fine-tuned this model and achieved 95.8%, 97.1%, and 85.35% accuracy for the HAM10000, ISBI2018, and ISBI2019 datasets, respectively. In comparison, another study [30] trained three different models (InceptionV3, ResNet, and VGG19) on a dataset containing 24,000 images retrieved between 2019 and 2020 from the ISIC archive. They concluded that InceptionV3 outperformed the rest of the models regarding accuracy. On the other hand, Khamparia et al. [31] incorporated transfer learning while training different deep learning architectures and proved that transfer learning and data augmentation helped to improve the results.

When training deep learning models, data imbalance is always an issue. There are many ways by which authors improve datasets by incorporating data augmentation techniques [32]. Ahmad et al. [33] used a data augmentation technique called generative adversarial networks (GAN), which creates artificial images similar to the original images to improve the dataset. With the help of this technique, they claim that their model accuracy was enhanced from 66% to 92%. In another study Kausar et al. [34] used some fine-tuning techniques to improve state-of-art deep learning image classification models. They achieved an accuracy of 72%, 91%, 91.4%, 91.7%, and 91.8% for ResNet, InceptionV3, DenseNet, InceptionResNetV2, and VGG-19, respectively. Khan et al. [35] proposed a multiclass deep learning model trained on the HAM10000, ISBI2018, and ISIC2019 datasets. They also incorporated transfer learning, and their results showed that the proposed model achieved an accuracy of 96.5%, 98%, and 89% for the HAM10000, ISBI2018, and ISIC2019 datasets, respectively. In another study, Deepa et al. [36] trained the ResNet50 model on the International Skin Image Collaboration (ISIC) dataset and achieved 89% accuracy. Tahir et al. [37] proposed a deep learning model called DSCC_Net, trained that on three datasets, ISIC 2020, HAM10000, and DermIS, and achieved an accuracy of 99%. They further compared their model with other state-of-art models and concluded that it outperformed them all. In another study, Shaheen et al. [38] proposed a multiclass model using particle swarm optimization trained on the HAM1000 dataset. They claim that their model achieved 97.82% accuracy.

As described above, there is a general trend for image processing based on deep learning to gradually adopt deeper networks. The benefit of using deeper networks is obvious, i.e., a deeper network provides stronger nonlinear representation capability. This means that, for some specific problems, a deeper network may be better able to learn more complex transformations and thus fit more complex feature inputs. However, previous research (see, e.g., [39]) has also shown ways in which network depth may negatively affect classification performance in cases where relatively simpler features are involved. Here, we first quantitatively assess the effect of network depth on classification performance and then develop a shorter and broader variant of the originally selected model (termed SBXception). The main contributions of this paper are the following:

- We analyze the characteristics of the adopted dataset (HAM10000) to show that network depth beyond an optimal level may not be suitable for classification tasks on this dataset.
- A new, shorter, broader variant of the Xception model is proposed to classify various skin lesions efficiently.
- The proposed modified model architecture is used to provide better classification performance compared to the state-of-the-art methods.

## 2. The Proposed Approach

**Figure 1:**
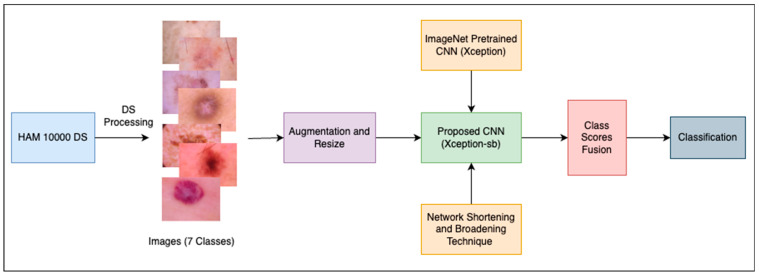
Overall architecture of the proposed framework.

This work proposes an approach to accurately classify skin lesions into seven classes pertaining to the most common types. The overall architecture of the approach, shown in Figure 1, involved three main stages. First, the dataset was prepared to make it more suitable for the classification task. Second, the effect of network depth on the classification performance was quantitatively explored, leading to the development of SBXception—a shorter and broader variant of the original model. Third, the proposed SBXception model was used for the classification task. In the following subsections, we provide a detailed discussion of the various stages involved in the development of the system.

### 2.1. Dataset and Input Images Preparation

In order to carry out the experiment, this work utilized a public dataset, the HAM10000 dataset [40]. This dataset is a widely-used collection of dermatoscopic images of skin lesions. It contains a total of 10,015 images acquired from individuals across various demographic regions. It includes images from different age groups, ethnicities, and geographical locations, providing a representative sample of skin conditions worldwide. Each image in the HAM10000 dataset includes a unique identifier (lesion_id) containing a 7-digit number corresponding to a unique patient record number. This allows each image to be linked to a single patient record. This way, the dataset enables researchers to accurately correlate an image with its respective patient, facilitating comprehensive analysis and longitudinal studies.

**Figure 2:**
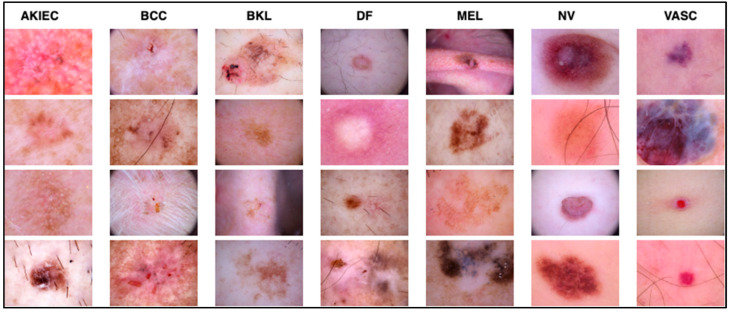
Samples of the seven types of diseases included in the HAM-10000 dataset.

The dataset contains images of seven classes of skin lesions, including actinic keratoses and intraepithelial carcinoma (AKIEC) (327), basal cell carcinoma (BCC) (514), benign keratosis (BKL) (1099), dermatofibroma (DF) (115), melanoma (MEL) (1113), melanocytic nevi (NV) (6705), and vascular skin lesions (VASC) (142). It is important to note that the original size of each image is 600 × 450. In contrast, in this experiment, the size was modified to 229 × 229 for efficient processing using the modified Xception architecture used in this research. Figure 2 shows some sample images of skin lesions. The same dataset was split into training, testing, and validation sets to ensure that the results were consistent.

### 2.2. Model Architecture

Existing image classification solutions increasingly use deeper neural networks as computing power improves and more solutions to the gradient disappearance problem become available. The benefit of this approach is self-evident, i.e., a deeper network provides stronger nonlinear representation capability. This means that, in general, a deeper network may be better able to learn more complex transformations and thus fit more complex feature inputs [41]. However, our experiments (reported in Section 3) revealed that a deeper network is not necessarily beneficial for the classification task on the HAM10000 dataset. Therefore, the current study modified the base model by decreasing its depth and increasing its width to better suit the given classification problem. The following subsections discuss this in more detail.

#### 2.2.1. The Base Model

**Figure 3:**
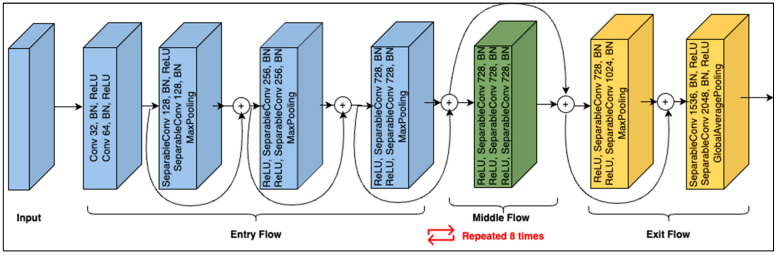
Xception module structure.

To gain insight into the data set, the initial experiments were conducted using the Xception [42] network. The structure of the Xception network is shown in Figure 3. As shown, its architecture is based on modified depth-wise separable convolution layers. The input has to go through three flows, i.e., the entry flow, middle flow (which repeats eight times), and exit flow. Each of the convolution and separable convolutional layers is followed by batch normalization. The middle flow is the core structural part of the Xception network, comprising a nine-layer structure that repeats eight times. Each of the nine layers in the structure contains three combinations of ReLU, separable Conv2D, and batch normalization layers.

#### 2.2.2. Shortening the Architecture

As shown in Figure 3, the core structure of Xception repeats eight times. Hence, it has a huge number of convolution layers. Network deepening often helps improve performance when dealing with images containing complex information, such as scenes containing human behavioral aspects, from which an exceedingly high number of features can be extracted. Network depth does not necessarily improve performance in several situations. For example, when the data set contains a limited number of objects with few details, see, e.g., [43], limited features will exist in the image, and consequently, the fault tolerance will be poor. Similarly, due to the very heavy optimization of gradient backpropagation, deep learning models tend to significantly overfit when the data are insufficient [44]. On the other hand, studies have shown that lowering the number of convolution layers has a significant impact on network performance; see, for example, [45,46]. Furthermore, limiting the number of convolution layers also has a significant impact on network performance in terms of computation efficiency [47,48]. Therefore, inspired by previous works [43,47], this study explored the relationship between network depth and its performance specifically using the HAM10000 dataset. To this end, we kept the network’s non-core (non-repeating) structure unchanged and experimented with repeating the core part for different numbers of times. Recall that each repetition in the core contains a combination (named RCB) of ReLU, separable Conv2D, and batch normalization layers. Therefore, by adopting a different number of RCB layers, seven modified forms of the Xception network (named Xception-mN) were created such that Xception-m1 contains one RCB, Xception-m2 has two RCBs, and so on, up to Xception-m7, which contains seven RCBs. To investigate the impact of network depth on classification accuracy, the performance of each of the modified networks was monitored. Here, the shortest network, i.e., Xception-m1 (with only one RCB layer), scored the highest in terms of accuracy and number of parameters. This showed that shortening the network enhanced the classification in terms of computational efficiency (decreased number of network parameters) as well as accuracy. Hence, it was concluded that classification of the HAM10000 dataset could not benefit from a deeper network. Next, we developed a network widening mechanism to further increase the classification performance.

#### 2.2.3. Broadening the Architecture

**Figure 4:**
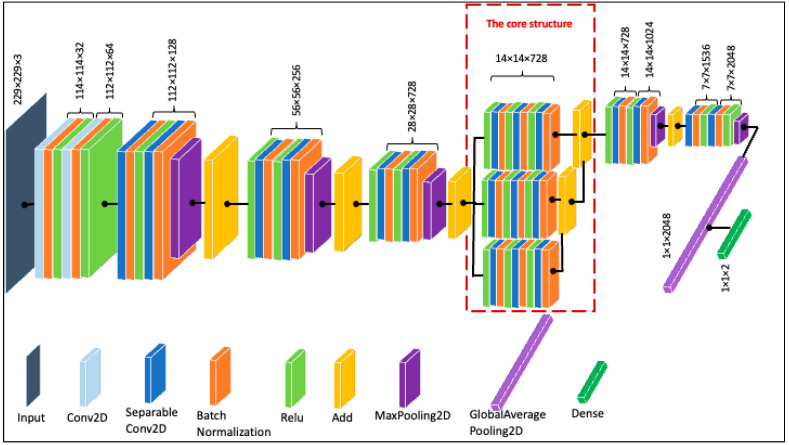
The proposed Xception structure (Xception-sb3).

Depth and breadth are two characteristics of a convolutional neural network that have the potential to affect its performance significantly. If the network has appropriate depth and width, it can learn a great deal of features and have higher nonlinear representational capabilities [49]. When optimizing the network structure, deepening the network is generally preferred over widening it, as it typically results in greater performance increases [50]. However, studies have found that once the network has reached a certain depth, adding further depth either makes the network harder to train with insignificant performance gains or, occasionally, causes its performance to degrade [39]. Similarly, several studies have shown that shallow and wide networks can achieve higher or at least as much accuracy as their deep and narrow counterparts [41,51,52]. Furthermore, in our initial experiments on the HAM10000 dataset, the shallowest Xception structure emerged as more suitable than the deeper alternatives. Thus, inspired by [43], we experimented with broadening the Xception network structure to improve its performance. The broadening mechanism essentially works by introducing a new add layer to obtain fusion of the horizontal channels by stacking the outputs of various branches. To achieve this, the network width can be increased in more than one way, such as by increasing the convolution layer channels or using a concatenate layer to connect the two expanded branches of the core structure. Here, we adopted a strategy similar to Shi et al. [43]. Specifically, we first expanded a branch of the core structure and then connected the output of two branches with the add layer. With this mechanism, there was no need to include a 1×1 convolution layer in the residual connection, because the number of channels before and after connection remained constant. Furthermore, we deemed it important to gauge the classification performance with different numbers of layer combinations in the broader architecture. Thus, by adopting different numbers of ReLU, Conv2D, and Batch Normalization (RCB) layers, eight shorter, broadened variations of the Xception network (named Xception-sbN) were created such that Xception-sb1 contains one RCB, Xception-sb2 contains two RCBs, and so on, up to Xception-sb8, which contains eight RCBs. The performance of each of the network architectures was monitored. The architecture with three RCBs, i.e., Xception-sb3 (shown in Figure 4), yielded the highest scores considering the accuracy and number of parameters. Therefore, this structure was used for the rest of the experiments, as detailed in the next section.

### 2.3. Fine-Tuning and Testing

For fine-tuning, the following HAM10000 splits were used, and the augmentation techniques described above were used. The dataset was split to use 80% for training and 20% for testing. Furthermore, 20% of the training dataset was used for validation. Table 1 shows the details of the numbers of images of each class used for training (before and after augmentation) and testing. The batch size was set to 32. The model’s performance was evaluated using diverse network configurations, employing various optimizers, learning rates, and momentum values. In cases where loss reduction was not evident for more than ten epochs, the learning rate was reduced by a factor of 1/10. The network configuration that produced the best results was adopted for testing, as well as for the remaining experiments, which will be discussed in the next section.

## 3. Experiments

We performed several experiments to evaluate the proposed approach in terms of its capability to correctly classify the various types of skin lesions as well as to compare it with existing methods. The classification system was implemented in Python using Keras with TensorFlow 2.0. The training and testing were carried out in the Google Colab environment with a GPU.

Three basic categories of experiments were performed. First, the ideal network configuration was selected based on several factors, such as the Xception structure and the layers in the core part. Second, various measures were used to assess the performance of the proposed model for the specific classification task. Third, the method’s effectiveness was evaluated compared to state-of-the-art skin lesion classification techniques.

### Performance Evaluation of the Proposed Approach

We tweaked the base Xception network architecture by reducing the depth and increasing the breadth of the middle flow of the network. We performed several experiments to analyze the effect of these changes in the depth and breadth of the neural network. As the original architecture deployed eight repetitions of the nine-layer structure of RCB layers described above, we measured the network’s performance with one (Xception-m1) to seven repetitions (Xception-m7) to analyze the impact of varying network depth. We measured the accuracy of these architectures for the seven types of skin lesions mentioned above. Table 2 shows the classification accuracy for these skin lesions given different network depths. The number of parameters for each architecture is also presented in the table. It can be seen that the shallowest network (Xception-m1), i.e., the network with one repetition of the nine RCB layers, achieved the highest classification accuracy. The only exception was for NV skin lesions, for which both Xception-m1 and Xception-m2 achieved the same classification accuracy. Intuitively, the number of parameters was also the least for the shallowest architecture, thus making it the most efficient among all architectures. Xception-m1 reduced the number of parameters by 54.27% compared to the base Xception architecture.

After identifying the optimal depth of the Xception architecture, we experimented with varying breadths of the architecture by adding concatenate layers. Table 3 shows classification accuracy for the modified architectures with one (Xception-sb1) to eight concatenate layers (Xception-sb8). The experimental results showed that Xception-sb3 and Xception-sb4 achieved comparable results. While Xception-sb3 achieved higher accuracy scores for BCC, DF, NV, and VASC, Xception-sb4 outperformed it for AKIEC and MEL classification accuracy. Both architectures produced the same accuracy for BKL. We selected Xception-sb3 because of the lower number of parameters. Xception-sb3 reduced the number of parameters by 38.76% compared to the base Xception architecture.

**Figure 5:**
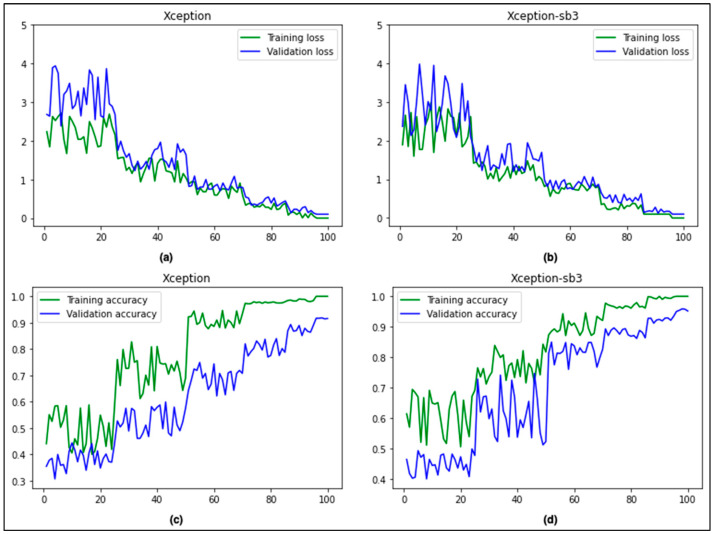
Comparison of accuracy and loss curves (Xception (**a**,**c**) vs. Xception-sb (**b**,**d**)).

Figure 5 compares the accuracy and loss curves between the base Xception and the proposed Xception-sb architectures. It is evident that our proposed technique produced slightly better results compared to the base architecture.

**Figure 6:**
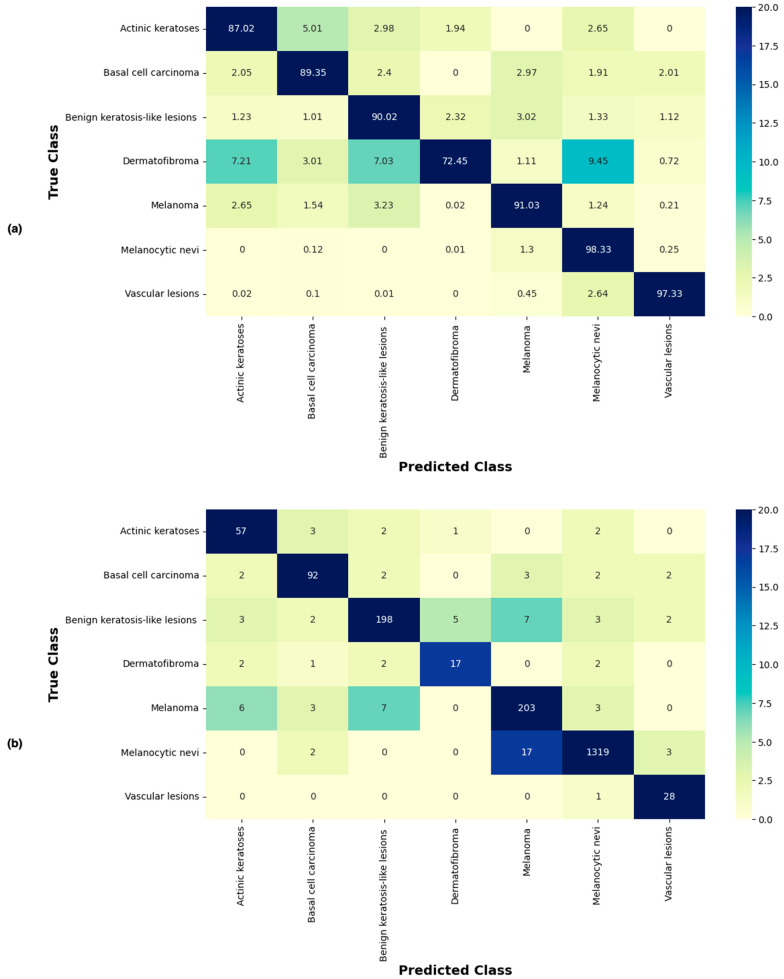
Confusion matrix of the proposed approach (using Xception-sb) showing predictions made for each class in terms of (**a**) percentages and (**b**) number of images of each class in the test set.

Figure 6 shows the confusion matrix of the proposed approach, showing predictions made for each class in terms of percentages and number of images from the test set correctly and incorrectly classified for each lesion type. It can be seen that the proposed technique correctly classified the highest number of images (i.e., 1319 of 1341) for the NV class, which had the most images to learn from. Images of VASC were also correctly classified at a high rate of 97.33%. MEL, BKL, BCC, and AKIEC lesions were correctly classified at rates of 91.03%, 90.02%, 89.35%, and 87.02%, respectively. With the lowest value of 72.45%, the correct classification of DF proved to be the most challenging for the proposed technique.

Table 4 shows recall, precision, accuracy, F1, and MCC (Matthew Correlation Coefficient) scores for each class of skin lesion. The proposed technique achieved the highest recall for the NV class (0.9832), while the lowest recall was recorded for DF (0.7175). On the other hand, the VASC class produced the best result for precision (0.9576). The NV class achieved the lowest precision (0.8365). The VASC class was classified with the highest accuracy (0.9893), and the lowest accuracy was recorded for the DF class (0.9532). The proposed technique produced high accuracy for all lesions in general. Finally, the best F1 score was achieved for the VASC class (0.9628), while the DF class was on the other side of the spectrum with the lowest score of 0.8153. As far as MCC scores are concerned, VASC yielded the best result (0.9565). MEL, NV, BCC, and AKIEC performed well, all scoring closely. DF scored the lowest MCC value (0.7989). Overall, the proposed technique produced the best classification results for the VASC class of lesions.

Table 4 also shows macro and weighted averages of recall, precision, accuracy, F1, and MCC scores to show the overall performance of the proposed technique. The proposed technique achieved a high overall level, with macro and weighted averages of 0.9689 and 0.9697, respectively. The overall recall of our approach was also recorded to be high, with macro and weighted averages of 0.8915 and 0.9543, respectively. The overall precision was calculated to comprise macro and weighted averages of 0.8946 and 0.8534, respectively. The macro and weighted average values for F1 score were measured to be 0.8899 and 0.8996, respectively. Finally, the respective macro and weighted average scores for MCC were calculated to be 0.8740 and 0.8848.

Table 5 compares the base Xception network architecture with the proposed optimal architectures regarding depth and breadth. Our depth-optimized Xception-m1 architecture outperformed the base architecture with a 0.83% improvement in accuracy, 54.27% improvement in the number of parameters, and 30.46% improvement in training time. Similarly, the proposed breadth-optimized Xception-sb3 architecture improved the accuracy of the base model by 2.63%, reduced the number of parameters by 38.77%, and resulted in a time reduction of 22.12% for training the network.

We also compared the proposed technique with state-of-the-art works in the problem domain. As shown in Table 6, our proposed technique outperforms the other works regarding accuracy and recall. The overall accuracy of our proposed approach was slightly better than the best results achieved by Naeem et al. [53]. We achieved significant improvement in recall compared to the existing works. Our overall recall of 0.9543 was about 2.38% better than the best recall achieved in previous studies. However, with a precision of 0.9292, Calderon et al. [54] still outperform all the existing methods, including our proposed technique.

## 4. Conclusions and Future Work

Skin cancer is considered one of the most serious and widespread health concerns worldwide, with a significant impact on patients’ quality of life and survival. The timely and accurate diagnosis of skin cancer is essential for effective treatment and improved outcomes. Deep learning models have shown considerable promise in assisting dermatologists with skin cancer diagnosis in recent years. In this study, we utilized a modified Xception model (called SBXception) to classify skin cancer lesions using the HAM10000 dataset. Our results demonstrated that SBXception, with its reduced and expanded architecture, had significantly improved performance in skin cancer classification, achieving an accuracy of 96.97% on a holdout test set. However, there are still some limitations to our study that need to be addressed in future research. Firstly, while our modified model achieved high accuracy on the HAM10000 dataset, its performance needs to be evaluated on other datasets to ensure its generalizability. Secondly, this study considered only the seven types of skin lesions found in the dataset. Additionally, the current work did not focus on the model’s interpretability to enhance its clinical applicability or other related factors, such as demographic bias.

In terms of future directions, one possible avenue for research is to explore the potential of combining multiple deep learning models for skin cancer diagnosis to further improve accuracy. Another future direction could be the development of a mobile application that can be created by adopting deep learning models to detect skin cancers. This application could provide an easy-to-access system for initial skin cancer diagnosis for people in remote areas. Additionally, a comprehensive dataset could be developed containing images from different populations and skin colors to ensure that the deep learning models can detect skin lesions from people with different colors.

## Figures and Tables

**Table 1:** Distribution of the HAM10000 dataset: training and testing splits by class.

Class	Total Images	Training (Original)	Training (Augmented)	Testing
AKIEC	327	262	5478	65
BCC	514	411	5168	103
BKL	1099	879	5515	220
DF	115	91	4324	24
MEL	1113	891	5088	222
NV	6705	5364	6306	1341
VASC	142	113	5317	29
Total	10015	8011	37197	2004

**Table 2:** Performance comparison of variations of the basic Xception structure.

Modified Structure	Accuracy							No. of Parameters
	AKIEC	BCC	BKL	DF	MEL	NV	VASC	
Xception	0.9413	0.9412	0.9323	0.9195	0.9444	0.9455	0.9520	20,873,774
Xception-m7	0.9413	0.9412	0.9323	0.9195	0.9444	0.9455	0.9520	19,255,430
Xception-m6	0.9430	0.9429	0.9340	0.9212	0.9461	0.9472	0.9537	17,637,086
Xception-m5	0.9449	0.9448	0.9359	0.9231	0.9480	0.9491	0.9556	16,018,742
Xception-m4	0.9422	0.9405	0.9321	0.9213	0.9482	0.9503	0.9569	14,400,398
Xception-m3	0.9470	0.9469	0.9380	0.9252	0.9501	0.9512	0.9577	12,782,054
Xception-m2	0.9501	0.9491	0.9421	0.9273	0.9532	0.9523	0.9599	11,163,710
**Xception-m1**	**0.9512**	**0.9498**	**0.9434**	**0.9273**	**0.9538**	**0.9531**	**0.9599**	**9,545,366**

**Table 3:** Performance comparison of variations of the Xception-sbN structure.

Modified Structure	Accuracy							No. of Parameters
	AKIEC	BCC	BKL	DF	MEL	NV	VASC	
Xception-sb8	0.9470	0.9523	0.9449	0.9328	0.9579	0.9572	0.9742	20,873,774
Xception-sb7	0.9558	0.9609	0.9547	0.9406	0.9669	0.9640	0.9821	19,255,430
Xception-sb6	0.9629	0.9678	0.9628	0.9467	0.9742	0.9691	0.9883	17,637,086
Xception-sb5	0.9630	0.9682	0.9633	0.9468	0.9744	0.9693	0.9887	16,018,742
Xception-sb4	**0.9635**	**0.9683**	**0.9634**	0.9472	**0.9751**	0.9701	0.9889	14,400,398
Xception-sb3	**0.9633**	**0.9685**	**0.9634**	**0.9532**	0.9747	**0.9702**	**0.9893**	**12,782,054**
Xception-sb2	0.9564	0.9548	0.9496	0.9315	0.9592	0.9563	0.9642	11,163,710
Xception-sb1	0.9512	0.9498	0.9434	0.9273	0.9538	0.9531	0.9599	9,545,366

**Table 4:** Classification results of the proposed approach.

Class	Recall	Precision	Accuracy	F1-Score	MCC-Score
AKIEC	0.8737	0.8686	0.9633	0.8712	0.8498
BCC	0.8874	0.8923	0.9685	0.8898	0.8714
BKL	0.8998	0.8519	0.9634	0.8752	0.8542
DF	0.7175	0.9441	0.9532	0.8153	0.7989
MEL	0.9110	0.9114	0.9747	0.9112	0.8965
NV	0.9832	0.8365	0.9702	0.9039	0.8905
VASC	0.9680	0.9576	0.9893	0.9628	0.9565
Macro Average	0.8915	0.8946	0.9689	0.8899	0.8740
Weighted Average	0.9543	0.8534	0.9697	0.8996	0.8848

**Table 5:** Comparison of accuracy, parameters, and training time.

Model	Accuracy (Weighted Average)	No. of Parameters	Training Time for Single Image (ms)
Xception	0.9434	20,873,774	44.71
Xception-m1	0.9517	9,545,366	31.09
Xception-sb3	0.9697	12,782,054	34.82

**Table 6:** Comparison of the classification accuracy with state-of-the-art works.

Reference	Pre-Training	Dataset	Recall	Precision	Accuracy
Calderon et al. [54]	ImageNet	HAM10000	0.9321	0.9292	0.9321
Jain et al. [55]	ImageNet	HAM10000	0.8957	0.8876	0.9048
Fraiwan and Faouri [56]	ImageNet	HAM10000	0.8250	0.9250	0.8290
Saarela and Geogieva [57]	-	HAM10000	-	-	0.8000
Naeem et al. [53]	ImageNet	ISIC 2019	0.9218	0.9219	0.9691
Alam et al. [58]	ImageNet	HAM10000	-	-	0.9100
Proposed method	ImageNet	HAM10000	0.9543	0.8534	0.9697

## Data Availability

The dataset used in this study is a public dataset [40].

## References

[1] WHO Key Facts about Cancer. https://www.who.int/news-room/fact-sheets/detail/cancer.

[2] Arora R., Raman B., Nayyar K., Awasthi R. (2021). Automated Skin Lesion Segmentation Using Attention-Based Deep Convolutional Neural Network. Biomed. Signal Process. Control.

[3] Esteva A., Kuprel B., Novoa R.A., Ko J., Swetter S.M., Blau H.M., Thrun S. (2017). Dermatologist-Level Classification of Skin Cancer with Deep Neural Networks. Nature.

[4] Kricker A., Armstrong B.K., English D.R. (1994). Sun Exposure and Non-Melanocytic Skin Cancer. Cancer Causes Control.

[5] Armstrong B.K., Kricker A. (2001). The Epidemiology of UV Induced Skin Cancer. J. Photochem. Photobiol. B.

[6] American Cancer Society. https://www.cancer.org/cancer/melanoma-skin-cancer/about/key-statistics.html.

[7] Larre Borges A., Nicoletti S., Dufrechou L., Nicola Centanni A. (2018). Dermatoscopy in the Public Health Environment. Dermatol. Public. Health Environ..

[8] Kasmi R., Processing K.M.-I.I. (2016). undefined Classification of Malignant Melanoma and Benign Skin Lesions: Implementation of Automatic ABCD Rule. Wiley Online Libr..

[9] Yu Z., Jiang F., Zhou F., He X., Ni D., Chen S., Wang T., Lei B. (2020). Convolutional Descriptors Aggregation via Cross-Net for Skin Lesion Recognition. Appl. Soft Comput..

[10] Celebi M.E., Kingravi H.A., Uddin B., Iyatomi H., Aslandogan Y.A., Stoecker W.V., Moss R.H. (2007). A Methodological Approach to the Classification of Dermoscopy Images. Comput. Med. Imaging Graph..

[11] Goel N., Yadav A., Singh B.M. (2020). Breast Cancer Segmentation Recognition Using Explored DCT-DWT Based Compression. Recent. Pat. Eng..

[12] Oliveira R.B., Pereira A.S., Tavares J.M.R.S. (2017). Skin Lesion Computational Diagnosis of Dermoscopic Images: Ensemble Models Based on Input Feature Manipulation. Comput. Methods Programs Biomed..

[13] Hosny K.M., Kassem M.A., Foaud M.M. (2019). Classification of Skin Lesions Using Transfer Learning and Augmentation with Alex-Net. PLoS ONE.

[14] Gulzar Y., Khan S.A. (2022). Skin Lesion Segmentation Based on Vision Transformers and Convolutional Neural Networks—A Comparative Study. Appl. Sci..

[15] Yélamos O., Braun R.P., Liopyris K., Wolner Z.J., Kerl K., Gerami P., Marghoob A.A. (2019). Usefulness of Dermoscopy to Improve the Clinical and Histopathologic Diagnosis of Skin Cancers. J. Am. Acad. Dermatol..

[16] Saba T., Khan M.A., Rehman A., Marie-Sainte S.L. (2019). Region Extraction and Classification of Skin Cancer: A Heterogeneous Framework of Deep CNN Features Fusion and Reduction. J. Med. Syst..

[17] Emanuelli M., Sartini D., Molinelli E., Campagna R., Pozzi V., Salvolini E., Simonetti O., Campanati A., Offidani A. (2022). The Double-Edged Sword of Oxidative Stress in Skin Damage and Melanoma: From Physiopathology to Therapeutical Approaches. Antioxidants.

[18] Ferlay J., Colombet M., Soerjomataram I., Parkin D.M., Piñeros M., Znaor A., Bray F. (2021). Cancer Statistics for the Year 2020: An Overview. Int. J. Cancer.

[19] American Cancer Society Cancer Facts and Figures. https://www.cancer.org/content/dam/cancer-org/research/cancer-facts-and-statistics/annual-cancer-facts-and-figures/2022/2022-cancer-facts-and-figures.pdf.

[20] Arbyn M., Weiderpass E., Bruni L., de Sanjosé S., Saraiya M., Ferlay J., Bray F. (2020). Estimates of Incidence and Mortality of Cervical Cancer in 2018: A Worldwide Analysis. Lancet Glob. Health.

[21] Australian Government Melanoma of the Skin Statistics. https://www.canceraustralia.gov.au/cancer-types/melanoma/statistics.

[22] Sung H., Ferlay J., Siegel R.L., Laversanne M., Soerjomataram I., Jemal A., Bray F. (2021). Global Cancer Statistics 2020: GLOBOCAN Estimates of Incidence and Mortality Worldwide for 36 Cancers in 185 Countries. CA Cancer J. Clin..

[23] Silverberg E., Boring C.C., Squires T.S. (1990). Cancer Statistics, 1990. CA Cancer J. Clin..

[24] Alam S., Raja P., Gulzar Y. (2022). Investigation of Machine Learning Methods for Early Prediction of Neurodevelopmental Disorders in Children. Wirel. Commun. Mob. Comput..

[25] Hamid Y., Elyassami S., Gulzar Y., Balasaraswathi V.R., Habuza T., Wani S. (2022). An Improvised CNN Model for Fake Image Detection. Int. J. Inf. Technol..

[26] Anand V., Gupta S., Gupta D., Gulzar Y., Xin Q., Juneja S., Shah A., Shaikh A. (2023). Weighted Average Ensemble Deep Learning Model for Stratification of Brain Tumor in MRI Images. Diagnostics.

[27] Haggenmüller S., Maron R.C., Hekler A., Utikal J.S., Barata C., Barnhill R.L., Beltraminelli H., Berking C., Betz-Stablein B., Blum A. (2021). Skin Cancer Classification via Convolutional Neural Networks: Systematic Review of Studies Involving Human Experts. Eur. J. Cancer.

[28] Khan S.A., Gulzar Y., Turaev S., Peng Y.S. (2021). A Modified HSIFT Descriptor for Medical Image Classification of Anatomy Objects. Symmetry.

[29] Khan M.A., Akram T., Sharif M., Kadry S., Nam Y. (2021). Computer Decision Support System for Skin Cancer Localization and Classification. Comput. Mater. Contin..

[30] Mijwil M.M. (2021). Skin Cancer Disease Images Classification Using Deep Learning Solutions. Multimed. Tools Appl..

[31] Khamparia A., Singh P.K., Rani P., Samanta D., Khanna A., Bhushan B. (2021). An Internet of Health Things-Driven Deep Learning Framework for Detection and Classification of Skin Cancer Using Transfer Learning. Trans. Emerg. Telecommun. Technol..

[32] Ayoub S., Gulzar Y., Rustamov J., Jabbari A., Reegu F.A., Turaev S. (2023). Adversarial Approaches to Tackle Imbalanced Data in Machine Learning. Sustainability.

[33] Ahmad B., Jun S., Palade V., You Q., Mao L., Zhongjie M. (2021). Improving Skin Cancer Classification Using Heavy-Tailed Student t-Distribution in Generative Adversarial Networks (Ted-Gan). Diagnostics.

[34] Kausar N., Hameed A., Sattar M., Ashraf R., Imran A.S., Ul Abidin M.Z., Ali A. (2021). Multiclass Skin Cancer Classification Using Ensemble of Fine-Tuned Deep Learning Models. Appl. Sci..

[35] Attique Khan M., Sharif M., Akram T., Kadry S., Hsu C.H. (2022). A Two-Stream Deep Neural Network-Based Intelligent System for Complex Skin Cancer Types Classification. Int. J. Intell. Syst..

[36] Deepa D., Muthukumaran V., Vinodhini V., Selvaraj S., Sandeep Kumar M., Prabhu J. (2023). Uncertainty Quantification to Improve the Classification of Melanoma and Basal Skin Cancer Using ResNet Model. J. Uncertain. Syst..

[37] Tahir M., Naeem A., Malik H., Tanveer J., Naqvi R.A., Lee S.W. (2023). DSCC_Net: Multi-Classification Deep Learning Models for Diagnosing of Skin Cancer Using Dermoscopic Images. Cancers.

[38] Shaheen H., Singh M.P. (2022). Multiclass Skin Cancer Classification Using Particle Swarm Optimization and Convolutional Neural Network with Information Security. J. Electron. Imaging.

[39] Zhang Z., Liu Q., Wang Y. (2018). Road Extraction by Deep Residual U-Net. IEEE Geosci. Remote Sens. Lett..

[40] Tschandl P., Rosendahl C., Kittler H. (2018). The HAM10000 Dataset, a Large Collection of Multi-Source Dermatoscopic Images of Common Pigmented Skin Lesions. Sci. Data.

[41] Shi J., Li Z., Ying S., Wang C., Liu Q., Zhang Q., Yan P. (2019). MR Image Super-Resolution via Wide Residual Networks with Fixed Skip Connection. IEEE J. Biomed. Health Inf..

[42] Chollet F. Xception: Deep Learning with Depthwise Separable Convolutions. Proceedings of the 30th IEEE Conference on Computer Vision and Pattern Recognition, CVPR 2017.

[43] Shi C., Xia R., Wang L. (2020). A Novel Multi-Branch Channel Expansion Network for Garbage Image Classification. IEEE Access.

[44] Hu G., Peng X., Yang Y., Hospedales T.M., Verbeek J. (2018). Frankenstein: Learning Deep Face Representations Using Small Data. IEEE Trans. Image Process..

[45] Liu Z., Chen Y., Chen B., Zhu L., Wu D., Shen G. (2019). Crowd Counting Method Based on Convolutional Neural Network with Global Density Feature. IEEE Access.

[46] Ding Y., Chen F., Zhao Y., Wu Z., Zhang C., Wu D. (2019). A Stacked Multi-Connection Simple Reducing Net for Brain Tumor Segmentation. IEEE Access.

[47] Mehmood A. (2021). Efficient Anomaly Detection in Crowd Videos Using Pre-Trained 2D Convolutional Neural Networks. IEEE Access.

[48] Mehmood A., Doulamis A. (2021). LightAnomalyNet: A Lightweight Framework for Efficient Abnormal Behavior Detection. Sensors.

[49] Montúfar G., Pascanu R., Cho K., Bengio Y. (2014). On the Number of Linear Regions of Deep Neural Networks. Adv. Neural Inf. Process Syst..

[50] Bengio Y., LeCun Y. (2007). Scaling Learning Algorithms towards AI. Large-Scale Kernel Mach..

[51] Chen L., Wang H., Zhao J., Koutris P., Papailiopoulos D. (2018). The Effect of Network Width on the Performance of Large-Batch Training. Adv. Neural Inf. Process Syst..

[52] Wu Z., Shen C., van den Hengel A. (2019). Wider or Deeper: Revisiting the ResNet Model for Visual Recognition. Pattern Recognit..

[53] Naeem A., Anees T., Fiza M., Naqvi R.A., Lee S.W. (2022). SCDNet: A Deep Learning-Based Framework for the Multiclassification of Skin Cancer Using Dermoscopy Images. Sensors.

[54] Calderón C., Sanchez K., Castillo S., Arguello H. (2021). BILSK: A Bilinear Convolutional Neural Network Approach for Skin Lesion Classification. Comput. Methods Programs Biomed. Update.

[55] Jain S., Singhania U., Tripathy B., Nasr E.A., Aboudaif M.K., Kamrani A.K. (2021). Deep Learning-Based Transfer Learning for Classification of Skin Cancer. Sensors.

[56] Fraiwan M., Faouri E. (2022). On the Automatic Detection and Classification of Skin Cancer Using Deep Transfer Learning. Sensors.

[57] Saarela M., Geogieva L. (2022). Robustness, Stability, and Fidelity of Explanations for a Deep Skin Cancer Classification Model. Appl. Sci..

[58] Alam T.M., Shaukat K., Khan W.A., Hameed I.A., Almuqren L.A., Raza M.A., Aslam M., Luo S. (2022). An Efficient Deep Learning-Based Skin Cancer Classifier for an Imbalanced Dataset. Diagnostics.

